# Unguarded Tricuspid Valve and Pulmonary Atresia With Intact Ventricular Septum Complicated With Right Coronary Artery Fistula and Advanced Atrioventricular Block in a Fetus: A Case Report

**DOI:** 10.7759/cureus.54209

**Published:** 2024-02-14

**Authors:** Hideharu Oka, Mio Taketazu, Rina Imanishi, Sorachi Shimada, Saori Sugiyama, Kentaro Nakanishi, Akiko Yoshizawa, Asako Kanai, Yuko Yokohama, Tomohiro Nawa, Madoka Sawada, Motoki Takamuro, Kouichi Nakau

**Affiliations:** 1 Pediatric Cardiology, Asahikawa Medical University, Asahikawa, JPN; 2 Pediatrics, Hokkaido Ryoikuen, Institution for Children/Adults with Severe Disabilities, Asahikawa, JPN; 3 Pediatrics, Asahikawa Medical University, Asahikawa, JPN; 4 Obstetrics and Gynaecology, Asahikawa-Kosei General Hospital, Asahikawa, JPN; 5 Obstetrics and Gynaecology, Asahikawa Medical University, Asahikawa, JPN; 6 Pediatric Cardiology, Hokkaido Medical Center for Child Health and Rehabilitation, Sapporo, JPN

**Keywords:** atrioventricular block, coronary fistula, uhl’s disease, pulmonary atresia with intact ventricular septum, unguarded tricuspid valve

## Abstract

The unguarded tricuspid valve is a rare and severe condition. When found in the fetus, they mostly undergo abortion or intrauterine death. The details of the fetal course in such cases are poorly understood. Here, we report a case of an unguarded tricuspid valve detected at 20 weeks of gestation who developed a complete atrioventricular block and survived in utero. The fetus also had pulmonary atresia with intact ventricular septum, Uhl's disease, hypoplastic right ventricle, noncompacted left ventricle, valvular aortic stenosis, and right coronary artery fistula to the right ventricle. Despite this serious condition, the fetal hydrops did not develop. The baby was born at 33 weeks of gestation but died on day two. Our experience suggests that some babies may survive the fetal period even with the severe type of an unguarded tricuspid valve. Hence, efficient fetal and neonatal treatment strategies for fetal unguarded tricuspid valves are crucial.

## Introduction

Unguarded tricuspid valve, a variant of tricuspid valve dysplasia, is a rare and severe condition. When found in the fetus, abortion or intrauterine death typically occurs [1,2]; therefore, the details of the fetal course are poorly understood [1-3]. However, there have been some cases found in adulthood, suggesting that the prognosis is not necessarily poor [4]. Here, we report a case with an unguarded tricuspid valve, pulmonary atresia with an intact ventricular septum, right coronary artery fistula to the right ventricle, and an advanced atrioventricular block who survived the fetal period.

## Case presentation

**Figure 1:**
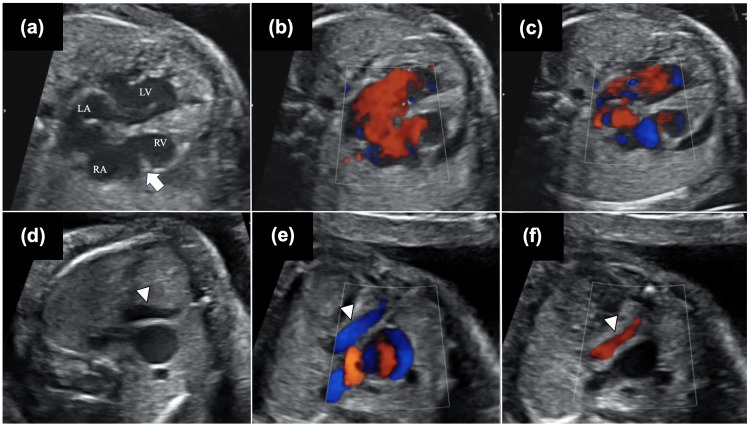
Fetal echocardiogram Four-chamber view from the fetal echocardiogram. (a) The tricuspid valve was not functioning (arrow), and the right ventricle was hypoplastic, with partial wall thinning and the morphology of Uhl's disease. (b, c) The right atrial-ventricular blood flow was to and fro (Figure 1b was a systolic phase, and Figure 1c was a diastolic phase). (d-f) The coronary artery fistula (arrowhead), misdiagnosed as the main pulmonary artery before birth, was located anterior to the aorta, displaying a to-and-fro flow on color flow mapping. RA, right atrium; RV, right ventricle; LA, left atrium; LV, left ventricle

A 31-year-old healthy woman was referred to us at 20 weeks’ gestation because of a suspicious association with fetal heart disease. Fetal echocardiography showed right atrial dilatation and hypoplastic right ventricle. The tricuspid valve was non-functional, and the hypoplastic right ventricle was associated with partial wall thinning, indicative of Uhl's disease (Figure 1a, Videos 1, 2). The right atrial-ventricular blood flow was to and fro (Figures 1b, 1c). While retrograde blood flow from the ductus arteriosus was observed in the bilateral pulmonary artery, the structure that could be clearly identified as the main pulmonary artery was not detected. A tubular structure with to-and-fro flow was observed in the anterior aspect of the right ventricle, suspected to be the main pulmonary artery (Figures 1d-1f, Movie 3). The left ventricle was hypertrophied, and the endocardium was coarse. The atrial rate was 140-200 bpm, and the ventricular rate was 70 bpm without atrioventricular conduction. We diagnosed this case as unguarded tricuspid valve and pulmonary atresia with intact ventricular septum, Uhl's disease, hypoplastic right ventricle, hypertrophied and non-compacted left ventricle, and complete atrioventricular block. The differential diagnosis of unguarded tricuspid valve includes Ebstein's anomaly and dysplastic tricuspid valve, but the findings of fetal echocardiography have ruled them out. No extracardiac complications were observed. The parent decided to continue the pregnancy despite being informed that the fetus was severely ill and that the prognosis could be poor. Counseling for the parents was provided at each weekly visit, with multidisciplinary involvement. Tricuspid regurgitation due to the unguarded tricuspid valve remained moderate, and fetal edema did not develop. Unfortunately, the baby was born by emergency cesarean section due to an early rupture of membranes at 33 weeks. Because of fetal bradycardia, it was difficult to confirm fetal well-being. It was also difficult to deliver the baby vaginally at any time due to limited medical staff, so a cesarean section was chosen. Postnatal echocardiography confirmed that the main pulmonary artery was not identified, and a right coronary fistula to the right ventricle was observed. This coronary fistula was misdiagnosed as the main pulmonary artery before birth. Although we did not perform chromosome testing, there were no findings in the postnatal appearance that would suggest chromosomal abnormalities. The infant had maintained respiration, but showed circulatory insufficiency due to bradycardia, and the decision was made to implement ventricular pacing. Pacing with a heart rate of 160 bpm was initiated after birth and successfully stabilized his vitals. However, a sudden cardiogenic shock at night required cardiopulmonary resuscitation. Extracorporeal membrane oxygenation was introduced, but he passed away at two days of age.

**Video 1:** Supplemental movie 1 Four-chamber view (B mode).

**Video 2:** Supplemental movie 2 Four-chamber view (color Doppler).

**Video 3:** Supplemental movie 3 Three-vessel view (color Doppler).

## Discussion

An unguarded tricuspid valve is characterized by complete or partial agenesis of the tricuspid valvular and subvalvular structures [5]. As the tricuspid valve ring is typically normal, it can be difficult to diagnose when the valve moves in a flap-like fashion. The typical color Doppler flow pattern can help diagnose this spectrum [3]. Dysplasia of the tricuspid valve causes the right atrium and ventricle enlargement, reducing anterograde blood flow to the pulmonary artery and inhibiting growth [3,5]. It is commonly associated with pulmonary atresia comprising 70% of unguarded tricuspid valve cases [6]. According to Kumar's report, only one of the 12 patients with an unguarded tricuspid valve had chromosomal abnormalities, which leads to speculation that there is little relation between chromosomal abnormalities and prognosis [6].

Interestingly, this is a severe case in which the patient had multiple congenital heart diseases, including the unguarded tricuspid valve, and developed a complete heart block, yet fetal hydrops did not progress. It is known that fetal hydrops are more likely to occur in cases of atrioventricular valve regurgitation and arrhythmias [7]. Whatever the cause, fetal hydrops are expected to occur due to increased venous pressure. We believe that fetal hydrops did not occur in this case because blood flow from the right atrium to the left through foramen ovale with 4 mm in diameter is enough to prevent venous obstruction and elevation of central venous pressure. The fact that the ventricular rate was not extremely low and output was maintained was also helpful.

We think that the cause of the infant's cardiogenic shock is as follows: birth releases the fetus from the 8-10 mmHg amniotic pressure, leading to a decrease of right atrial and ventricular pressures [7]. Conversely, release from the placenta increases aortic pressure. These factors may lead to a critical boost in the coronary steal volume from the right coronary artery fistula to the right ventricle. Additionally, a decrease in right atrial and ventricular pressure may increase the circular shunt, where blood flows from the right coronary to the right ventricle, right ventricle to the right atrium, right atrium to the left atrium, left atrium to the left ventricle, left ventricle to the aorta, and aorta to the right coronary. Regarding pacing for bradycardia, due to the high pacing rate of 160 bpm, the diastolic pressure in the left ventricle may not decrease sufficiently, and the left ventricular diastolic period should be shortened. These could reduce coronary artery flow back to the left ventricle, potentially causing left ventricular ischemia. In this particular patient, a lower pacing rate may be more suitable to maintain adequate coronary supply. In patients with left ventricular hypertrophy, control of postnatal heart rate also needs to be thoroughly studied beforehand.

When an unguarded tricuspid valve is complicated by pulmonary atresia with intact ventricular septum or Uhl’s disease, maintaining an adequate right ventricular volume or function is challenging, and postnatal treatment strategies are essential. Right ventricular plication may be necessary in some situations to achieve single ventricle hemodynamics. In this case, due to the complication from the coronary artery fistula, the tricuspid valve might have needed to be closed to prevent coronary steal. Additional right ventricular plication might also have been required for future single ventricular repair.

## Conclusions

We encountered a case of an unguarded tricuspid valve diagnosed in utero. Despite critical tricuspid valve regurgitation and complete heart block, the baby was born without progression of fetal edema. However, it was difficult to save his life. Our experience suggests that some babies may survive the fetal period despite a diagnosis of a critical type of unguarded tricuspid valve. Further studies are needed to improve fetal and neonatal treatment quality and efficacy in cases of fetal unguarded tricuspid valve.
